# Eg5 Overexpression Is Predictive of Poor Prognosis in Hepatocellular Carcinoma Patients

**DOI:** 10.1155/2017/2176460

**Published:** 2017-06-08

**Authors:** Can Liu, Nan Zhou, Jieying Li, Jun Kong, Xi Guan, Xudong Wang

**Affiliations:** ^1^Department of Oncology, Affiliated Hospital of Nantong University, Nantong, Jiangsu 226001, China; ^2^Department of Pathology, Affiliated Hospital of Nantong University, Nantong, Jiangsu 226001, China; ^3^Center of Clinical Laboratory Medicine, Affiliated Hospital of Nantong University, Nantong, Jiangsu 226001, China

## Abstract

Eg5 (kinesin spindle protein) plays an essential role in mitosis. Inhibition of Eg5 function results in cell cycle arrest at mitosis, which leads to cell death. To date, Eg5 expression and its prognostic significance have not been studied in hepatocellular carcinoma (HCC). In this study, 26 freshly frozen HCC tissue samples and matched peritumoral tissue samples were evaluated with a one-step qPCR test and immunohistochemical (IHC) analysis was conducted on 156 HCC samples to investigate the relationships among Eg5 expression, clinicopathological factors, and prognosis. Eg5 mRNA and protein expression levels were significantly higher in HCC tissues relative to matched noncancerous tissues (*p* < 0.05). High Eg5 protein expression was significantly related to liver cirrhosis (*p* = 0.038) and TNM stage (*p* = 0.008). Kaplan-Meier survival and Cox regression analyses revealed that Eg5 overexpression (*p* = 0.001), liver cirrhosis (*p* = 0.009), and TNM stage (*p* = 0.025) were independent prognostic factors for overall survival. These findings indicate that Eg5 expression can be used as a biomarker of poor prognosis and as a novel therapeutic target for HCC.

## 1. Introduction

Globally, liver cancer, especially hepatocellular carcinoma (HCC), is the sixth most frequently diagnosed cancer and shows the third highest mortality rate in cancer patients [1, 2]. The highest incidences of HCC have been found in Asia, and increasing incidence rates have been reported in the USA and Europe [3]. Current treatments for liver cancer are varied and include surgery, targeted therapy, interventional radiology, and traditional treatment. For HCC, surgical resection remains the most common treatment, but despite operative treatment, the risk of recurrence is still high, affecting the long-term recovery of HCC patients [4]. Liver cancer has a long latency period, and most HCC patients are at an advanced stage and are often metastatic when diagnosed; for such patients, chemotherapy is a commonly used choice. Yet, conventional chemotherapy is less effective and is associated with severe side-effects [4, 5]. Therefore, there is an urgent need to develop new therapeutic targets for HCC. So far, numberless efforts have been made to find out reliable prognostic biomarkers for HCC, and corresponding targeted drug therapy has shown an association with a favorable HCC prognosis. These markers include angiogenesis-related factors (e.g., VEGF, PDGF) [6, 7], epithelial growth factor receptor (EGFR) [8], and mitogen-activated protein kinase (MAPK) [9]. Therefore, it is necessary to make efforts to find further high-quality HCC prognostic markers.

Eg5 (also known as Kiff11 or kinesin spindle protein) is a member of the kinesin-5 family and plays a crucial role in the formation and maintenance of the bipolar spindle during mitosis. Additionally, Eg5 has been regarded as an attractive target for cancer therapies [10]. Blocking Eg5 expression impairs the separation of duplicated centrosomes, leading to cell-cycle arrest in mitosis with monastrol microtubule arrays and triggering apoptotic cell death in tumor cells [11]. Indeed, recent studies have reported that Eg5 expression is associated with several malignancies, such as hepatic carcinoma [12], lung cancers [13], pancreatic cancer [14], gastric cancer [15], colorectal cancer [16], and prostate cancer [17]. Since the first Eg5 inhibitor, monaster, was identified in 1999 [18], a number of Eg5 inhibitors have been discovered and used in research [16, 19]. Eg5 is overexpressed in tumors relative to normal tissues, and it is not found in postmitotic human neurons, making it an attractive target for novel anticancer therapies [20].

Although the expression of Eg5 has been associated with prognosis in certain tumor types, few studies have explored the relationship between Eg5 and liver cancer. In the present report, we studied Eg5 expression in 299 HCC samples and tumor-adjacent tissues. Furthermore, we evaluated the importance and clinical pathological significance of Eg5 in cancer patients with HCC.

## 2. Materials and Methods

### 2.1. HCC Sample Collection

Fresh-frozen tumor (26 HCC) and matched noncancerous tissue samples (*n* = 26) were collected from the Department of Pathology at the Hospital of Nantong University. Simultaneously, 156 formalin-fixed, paraffin-embedded HCC tumor specimens, 69 matched tumor-adjacent tissues, and 74 benign samples (cirrhosis of liver, 28 cases; liver hemangioma, 12 cases; focal nodular hyperplasia, 10 cases; intrahepatic bile duct dilatation, 9 cases; cholecystitis, 8 cases; and cyst of liver, 7 cases) were also collected from the Department of Pathology at the Hospital of Nantong University, from 2006 to 2016. Histological diagnosis of HCC was performed according to the latest WHO criteria [21]. All HCC patients were histopathologically determined by 2 pathologists. No patients received immunotherapy, chemotherapy, or radiotherapy before their surgical operation. The patient ages ranged from 31 to 79 years, with a mean age of 55 years. Survival time was defined as the interval from the date of operation to the date of death. Clinical data (including gender, age, histological type, TNM stage, *α*-fetoprotein (AFP) serum level, tumor size, differentiation, and vascular invasion) and other information were retrospectively collected from each patient's records. The protocol for the present study was acquired by the Research Ethics Committee of the Affiliated Hospital of Nantong University. Twenty-six tissue blocks from patients with malignancies and with 5 years of follow-up records were also available, and these were used to construct the tissue microarray (TMA).

### 2.2. One-Step qPCR Test

Total RNA was isolated from 26 freshly frozen HCC specimens and from matched peritumoral tissues using TRIzol reagent (Invitrogen, USA). Immediately after extraction, the RNA quantity and quality were analyzed by spectrophotometry. The RNA was then reverse-transcribed into cDNA with Moloney murine leukemia virus reverse transcriptase (Promega, USA). One-step PCR was performed as previously reported [22]. Eg5-specific oligonucleotide primers (forward 5′-GAA CAA TCA TTA GCA GCA GAA-3′ and reverse 5′-TCA GTA TAG ACA CCA CAG TTG-3′) were designed to yield a 118 bp PCR product. Eg5 gene expression was standardized against *β*-actin expression (forward primer 5′-TAA TCT TCG CCT TAA TAC TT-3′ and reverse primer 5′-AGC CTT CAT ACA TCT CAA-3′) (Supplementary Table available online at https://doi.org/10.1155/2017/2176460). The liver cancer of TCGA data was downloaded from https://genome-cancer.ucsc.edu/proj/site/hgHeatmap/.

### 2.3. Immunohistochemical (IHC) Staining

A total of 156 HCC tissue samples, 74 matched adjacent liver tissue samples, and 69 benign tissue samples were collected and used in this study. TMAs were assembled using a tissue array instrument (Quick-Ray, UT06, UNITMA, Korea). All tissues were formalin-fixed and paraffin-embedded, and the TMA samples were cut into 4 *μ*m sections and placed on Superfrost charged glass microscope slides.

The TMA microarray sections were separately stained with anti-Eg5 antibody (1 : 100; Abcam, USA) overnight at 4°C, washed with PBS, incubated with a biotinylated secondary antibody for 15 min and washed again. The sections were stained with 3,3-diaminobenzidine (DAB) chromogen solution for 15 min, and the sections were then counterstained with hematoxylin. For negative controls, PBS was used in place of the primary antibody. In the present study, EG5 immunostaining for each slide was scored by two independent pathologists according to the staining intensity and the percentage of EG5-positive cells at that intensity. Percentage of Eg5 gene-positive cells were scored as follows: 0 (0% tumor cells stained), 1 (1–33% tumor cells stained), 2 (34–66% tumor cells stained), and 3 (67–100% tumor cells stained). Additionally, the Eg5 staining intensities were scored as follows: 0 (+, negative), 1 (+, weakly positive), 2 (++, moderately positive), and 3 (+++, strongly positive). The final EG5 gene staining score was the sum of the intensity and percentage scores, and samples with a summed score of less than 2 were defined as exhibiting low expression, while those with a summed score of 3–6 were defined as exhibiting high expression [23, 24].

### 2.4. Statistical Methods

The Eg5 mRNA expression levels in the freshly frozen HCC and matched normal tissues were analyzed using the nonparametric Wilcoxon signed-rank test. The relationships between Eg5 expression levels in the tumor samples and patient clinicopathological features were evaluated using the *χ*^2^ test. The survival rate was calculated with the Kaplan-Meier and log-rank tests. The TCGA data for liver cancer was downloaded from https://cancer-genome.ucsc.edu. Univariate and multivariate analyses were performed using the Cox hazard regression method. *p* values of less than 0.05 were considered to be statistically significant. Data were analyzed using STATA 12.0 software (Stata Corporation, USA).

## 3. Results

### 3.1. Eg5 mRNA Expression in HCC and Peritumoral Tissues

**Figure 1:**
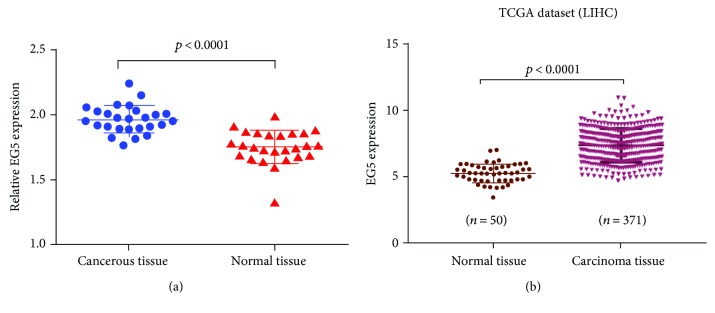
Eg5 mRNA expression in HCC and normal tissues. (a) One-step qPCR was performed to compare Eg5 mRNA expression levels in cancerous tissues with those in adjacent normal tissues. The Eg5 mRNA level in the HCC tissue (1.976 ± 0.02068) was higher on average than that in the matched adjacent tissue. *β*-Actin was used as an internal control. (b) In TCGA dataset, the Eg5 mRNA level in the carcinoma tissue was higher on average than that in the matched normal tissue (*p* < 0.0001).

To investigate Eg5 mRNA expression in HCC patients, RNA was extracted from 26 cancerous and 26 adjacent tissues and analyzed using one-step qPCR. When comparing Eg5 expression in cancerous and noncancerous tissues, we found that Eg5 mRNA expression was significantly higher in the HCC tissues than in the normal tissues (1.967 ± 0.020 versus 1.754 ± 0.024, resp.; *t* = 6.559, *p* < 0.0001) (Figure 1(a)). And in TCGA dataset, 371 cancerous tissues and 50 noncancerous liver tissues were detected using HiSeq; the expression of Eg5 mRNA in normal and carcinoma tissues has significant statistical differences (*p* < 0.0001) (Figure 1(b)).

### 3.2. Eg5 Protein Expression in HCC Tissues Detected by IHC

**Figure 2:**
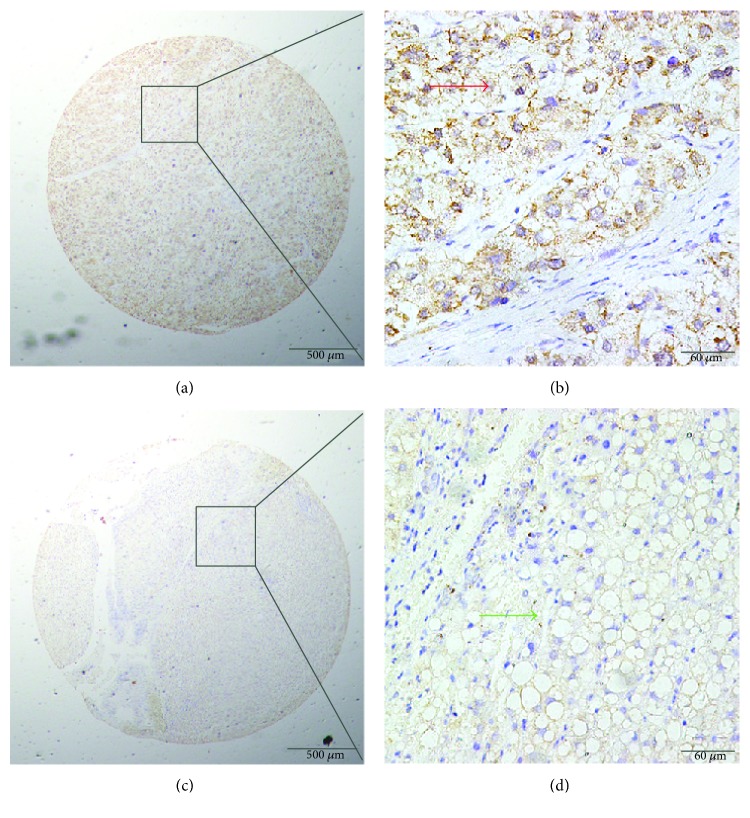
Immunohistochemical (IHC) staining for Eg5 expression in cancerous and peritumoral tissues. (a, b) Positive cytoplasmic IHC staining (red arrow) for Eg5 in HCC tissue samples. (c, d) Negative IHC staining (green arrow) for Eg5 in normal tissue samples (original magnification: ×40 in (a) and (c); ×400 in (b) and (d)).

We studied Eg5 expression in the HCC patient samples using IHC with a TMA (156 HCC samples, 69 matched peritumoral samples, and 74 benign liver cancer tissue samples). Typical IHC staining patterns for Eg5 are shown in Figure 2. Positive staining for Eg5 was predominantly localized to the cytoplasm of HCC cells; however, in some samples, positive Eg5 staining was also observed at the cell membranes. The results showed that Eg5 expression was markedly high in the HCC tumors, but low or negative in benign or adjacent liver tissue (Figure 2). Among the HCC tumors, high cytoplasmic expression of Eg5 was found in 67.31% (105/156) of the liver cancer samples, compared with only 43.48% (30/69) of benign tissues and 47.30% (35/74) of peritumoral tissues. We determined the cutoff point for Eg5 to be 150 using the X-tile software program (the Rimm Lab at Yale University; http://medicine.yale.edu/lab/rimm/research/software.aspx), and the data represented statistical significance (*χ*^2^ = 14.738, *p* < 0.001) (Table 1).

### 3.3. Relationship between Eg5 Expression and Clinicopathological Features in HCC Samples

To confirm the important role of Eg5 in liver cancer progression, we summarized the relationship between Eg5 expression and the major clinicopathological variables of HCC in Table 2. Stratifying the clinical parameters by the two Eg5 expression levels, we found that Eg5 expression was significantly associated with TNM stage (*p* = 0.008) and liver cirrhosis (*p* = 0.038) (Table 2). However, we found no statistically significant association between high Eg5 expression and age, gender, liver cirrhosis, differentiation, gross classification, hepatitis B virus infection, vascular invasion, or AFP levels (Table 2).

### 3.4. Eg5 Overexpression Predicts Poorer Prognosis

**Figure 3:**
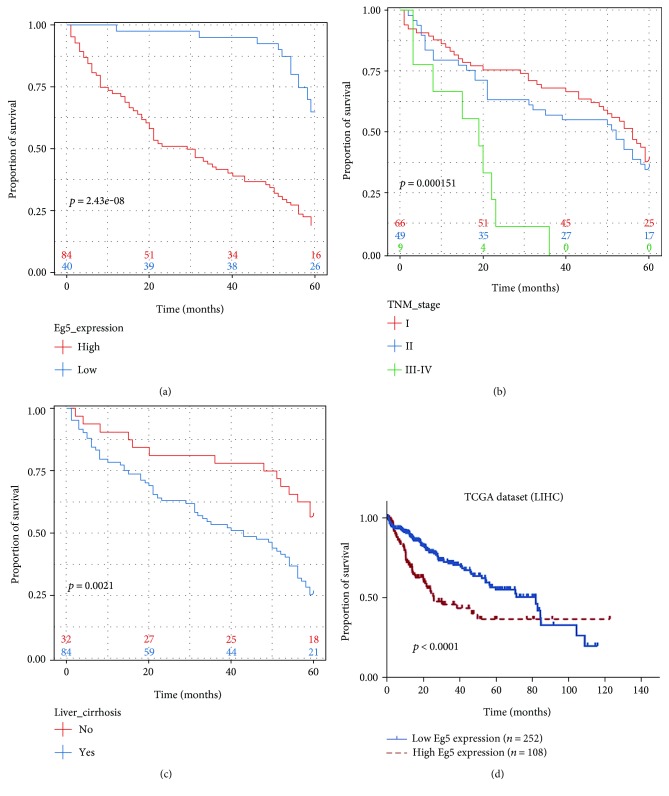
Kaplan-Meier survival curves of HCC patients after surgical therapy. (a) Patients with high Eg5 expression levels (red line) exhibited lower overall survival rates than patients with low Eg5 expression levels (blue line). (b) Patients at an advanced TNM stage (stages III-IV) (green line) exhibited a statistically lower overall survival rate than those at an early TNM stage. (c) Patients with liver cirrhosis (blue line) exhibited poor prognoses compared with patients without liver cirrhosis. (d) In TCGA dataset, patients with high Eg5 expression levels (red line) exhibited lower overall survival rates than patients with low Eg5 expression levels (blue line).

Using univariate Cox regression analysis, we found that high Eg5 expression was significantly associated with inferior outcome in HCC patients (*p* = 0.001). Additionally, other HCC clinical prognostic factors, such as liver cirrhosis (*p* = 0.003), gross classification (*p* = 0.007), and TNM stage (*p* = 0.011), were significantly correlated with a decreased 5-year survival rate (Table 3). Factors that were independent of prognostic factors in univariate models were assessed in the multivariate Cox regression analysis. Moreover, our data showed that high Eg5 expression (*p* = 0.001), liver cirrhosis (*p* = 0.009), and TNM stage (*p* = 0.025) were all significant predictors of survival in HCC. Kaplan-Meier survival analysis also demonstrated that HCC patients with high Eg5 expression, advanced TNM stage, or liver cirrhosis had significantly shorter survival times (Figures 3(a), 3(b), and 3(c)). Simultaneously, in TCGA dataset, patients with low Eg5 expression levels exhibited higher overall survival rates than patients with high Eg5 expression levels (Figure 3(d)).

## 4. Discussion

Eg5, a microtubule-based motor of the kinesin family, is considered to have a key role in the formation and maintenance of the bipolar spindle during mitosis and has been regarded as a key target of anticancer treatment [10, 25, 26]. In addition to its role in mitosis, an increasing number of studies have revealed other possible biological functions of Eg5. Liu et al. reported that Eg5 is involved in an immune regulation process; Eg5 interacts with Tat, which blocks cell-cycle progression in CD4-positive T lymphocytes, thereby leading to apoptosis [27]. Groth-Pedersen et al. observed that Eg5 siRNAs inhibit lysosomal trafficking and induce lysosomal membrane permeabilization by increasing lysosomal volume [28]. Additionally, Eg5 also plays important roles in protein translation, neuronal survival, cancer cell migration, and angiogenesis impairment [29–33]; therefore, Eg5 is believed to be a potential therapeutic target for solid tumors. High Eg5 expression has been reported in different types of malignancies [12–17]. However, to our knowledge, associations between Eg5 expression level and clinical parameters in HCC to evaluate the significant clinicopathological role of Eg5 have not yet been investigated.

In the present study, the qRT-PCR data showed that the mean Eg5 mRNA expression level in cancerous tissue was higher than that in the corresponding normal tissue, in accord with the results of IHC analysis in HCC samples. TMA and IHC analyses of the HCC tissues showed that the high Eg5 protein expression was mainly observed in the tumor cell cytoplasm, in agreement with previous reports [23]. Positive Eg5 protein staining was indicated in 67.31% (105/156) of the HCC tissue samples, representing an increase over the adjacent liver and benign liver disease tissues. These results verified our hypothesis and were in accord with previous reports indicating that high Eg5 protein expression levels are present in cancer tissue samples, such as in 60.58% of laryngeal squamous cell carcinoma [23] and 51.8% of renal cell carcinoma (RCC) [34]. Differences among studies may be due to the cutoff points that were chosen. Overall, the results support the conclusion that Eg5 expression levels in HCC cells are higher than those in normal hepatic cells. However, in our study, the expression of Eg5 in benign samples is relatively higher (47.30%, 35/74); this result might be due to 74 cases of benign tissues containing 28 cases of cirrhosis, 12 cases of liver hemangioma, and 10 cases of focal nodular hyperplasia. In the histopathology, liver cirrhosis has hepatocellular nodular regeneration and connective tissue hyperplasia; hepatic hemangioma with vascular endothelial abnormalities and focal nodular hyperplasia have fibrous tissue and small bile duct hyperplasia. Moreover, it may be that, in the present study, the number of benign samples is relatively insufficient.

We discovered a correlation between Eg5 expression, clinicopathological parameters, and prognosis. High Eg5 expression was correlated with TNM stage (*p* = 0.008); this result verified the pro-oncogenic role of Eg5 and is in agreement with reports that suggested a tumorigenic role in malignancies [23, 34, 35]. Additionally, we found the overexpression of Eg5 is related to liver cirrhosis (*p* = 0.038). EG5 is a microtubule-based motor of the kinesin family; it has a key role in the formation and maintenance of the bipolar spindle during mitosis. Therefore, the result may be owning to cirrhosis of the liver with extensive residual hepatocellular nodular regeneration and connective tissue hyperplasia. Furthermore, univariate and multivariate analyses indicated that increased Eg5 expression, advanced TNM stage, and liver cirrhosis are independent factors for unfavorable overall survival of HCC patients. Finally, our data clearly suggest that high Eg5 expression is significantly associated with poor HCC outcomes.

Interestingly, Oncomine data suggested that Eg5 transcripts are not overexpressed in kidney cancer. However, recent evidence has shown that, compared to that in normal tissue, higher Eg5 expression in RCC tumors leads to poor disease outcomes [34], consistent with the results of our study. Additionally, data from a lung cancer study showed that patients with high Eg5 expression had a higher response rate to chemotherapy than patients with low Eg5 expression (37% versus 10%) [13]; this opposite result might be due to differences in tumor types or in the internal environments of the patients. Moreover, Eg5 might possess multiple roles in cancer. A previous research study showed that SHIP2 (SH2-containing inositol 5-phosphatase 2) possesses both anticancer activities and oncogenic functions [36]. Therefore, our research requires further validation with a larger sample size, and we should perform in vitro and in vivo studies to verify the roles of Eg5 in HCC development.

The present study is the first to demonstrate the important role for Eg5 in HCC, not only at the mRNA level but also at the protein level. Our results provide evidence that Eg5 overexpression is related to poorer survival in HCC patients, which may be helpful in future research studies aimed at understanding the molecular mechanisms that underlie the development of HCC. Our findings have established the potential clinical value of Eg5 as a novel biomarker in HCC, and targeting Eg5 might provide a novel strategy for HCC treatment.

## 5. Conclusions

The expression of Eg5 in HCC was markedly higher when compared to that in the matched noncancerous tissues, and this overexpression was significantly related to liver cirrhosis (*p* = 0.038), advanced TNM stage (*p* = 0.008), and a poor prognosis. Overall, these findings suggest that Eg5 expression can be used as a biomarker of poor prognosis and even a novel therapeutic target for HCC.

## Supplementary Material

Supplementary Table. RT-PCR Primers.

## Figures and Tables

**Table 1:** Eg5 IHC staining in benign, cancerous, and adjacent liver tissues.

Tissue sample	*n*	Eg5 expression, *n* (%)		Pearson *χ*^2^	*p*
		Low or none	Positive		
Liver tissue with benign disease	74	39 (52.70)	35 (47.30)	14.738	0.001^∗^
Adjacent liver tissue	69	39 (56.52)	30 (43.48)		
Liver cancer	156	51 (32.69)	105 (67.31)		

^∗^ *p* < 0.05.

**Table 2:** Patient clinicopathological characteristics according to the Eg5 score.

Characteristic	*n*	Eg5 expression, *n* (%)		Pearson *χ*^2^	*p*
		Negative	Positive		
Total	156	51 (32.69)	105 (67.31)		
Gender				0.132	0.717
Male	119	38 (31.93)	81 (68.07)		
Female	37	13 (35.14)	24 (64.86)		
Age				1.867	0.172
<60	109	39 (35.78)	70 (64.22)		
≥60	45	11 (24.44)	34 (75.56)		
Unknown	2				
Differentiation				2.092	0.351
Well	12	6 (50.00)	6 (50.00)		
Middle	113	34 (30.09)	79 (69.91)		
Poor	31	11 (35.48)	20 (64.52)		
Liver cirrhosis				4.298	0.038^∗^
Yes	99	26 (26.26)	73 (73.74)		
No	46	20 (43.48)	26 (56.52)		
Unknown	11				
Gross classification				0.093	0.760
Solitary	139	46 (33.09)	93 (66.91)		
Multiple	17	5 (29.41)	12 (70.59)		
Hepatitis B viral infection				1.115	0.291
Yes	12	2 (16.67)	10 (83.33)		
No	128	39 (30.47)	89 (69.53)		
Unknown	16				
Vascular invasion				2.121	0.145
Yes	68	18 (26.47)	50 (73.53)		
No	88	33 (37.50)	55 (62.50)		
Tumor diameter				1.974	0.160
<5 cm	93	35 (37.63)	58 (62.37)		
≥5 cm	60	16 (26.67)	44 (73.33)		
Unknown	3				
TNM stage				9.710	0.008^∗^
I	79	30 (37.97)	49 (62.03)		
II	66	21 (31.82)	45 (68.18)		
III	11	0 (0.00)	11 (100.00)		
*α*-Fetoprotein (ng/ml)				0.959	0.327
<20	51	13 (25.49)	38 (74.51)		
≥20	68	23 (33.82)	45 (66.18)		
Unknown	37				

^∗^ *p* < 0.05.

**Table 3:** Univariate and multivariate analyses of prognostic factors for overall survival in liver cancer patients.

	Univariate analysis				Multivariate analysis			
	HR	*p* > |*z*|	95% CI		HR	*p* > |*z*|	95% CI	
Eg5 expression								
High versus low	4.490	<0.001^∗^	2.511	8.030	3.913	<0.001^∗^	2.136	7.167
Age (years)								
≤60 versus >60	0.983	0.949	0.589	1.643				
Gender								
Male versus female	0755	0.260	0.463	1.323				
Liver cirrhosis								
Yes versus no	2.396	0.003^∗^	1.339	4.286	2.169	0.009^∗^	1.210	3.889
Gross classification								
Solitary versus multiple	2.292	0.007^∗^	1.254	4.188				
Differentiation								
Well versus middle versus poor	1.217	0.353	0.804	1.843				
Hepatitis B viral infection								
Yes versus no	1.489	0.438	0.544	4.072				
Vascular invasion								
Yes versus no	1.132	0.582	0.728	1.761				
Tumor diameter (cm)								
<5 versus ≥5	1.287	0.277	0.816	2.029				
TNM stage								
I versus II versus III	1.604	0.011^∗^	1.112	2.313	1.524	0.025^∗^	1.053	2.206
Preoperative *α*-fetoprotein (ng/ml)								
<20 versus ≥20	0.900	0.658	0.564	1.436				

^∗^ *p* < 0.05; HR: hazard ratio; 95% CI: 95% confidence interval.
