# Supplementary material for: Eg5 Overexpression Is Predictive of Poor Prognosis in Hepatocellular Carcinoma Patients
Source: Dis Markers. 2017 Jun 8;2017:2176460. doi: 10.1155/2017/2176460 (PMC5480051; doi:10.1155/2017/2176460)
Supplement: Supplementary file 1 — Supplementary Table. RT-PCR Primers. [file 2176460.f1.pdf]

Supplementary Table . RT-PCR Primers

Gene	Primer sequences
**Eg5**	F: 5'-GAA CAA TCA TTA GCA GCA GAA-3 '
	R: 5'-TCA GTA TAG ACA CCA CAG TTG-3'
**β-actin**	F: 5' -TAA TCT TCG CCT TAA TAC TT-3'
	R: 5'-AGC CTT CAT ACA TCT CAA-3'
